# Hepatitis B and C virus infection and diabetes mellitus: A cohort study

**DOI:** 10.1038/s41598-017-04206-6

**Published:** 2017-07-04

**Authors:** Yun Soo Hong, Yoosoo Chang, Seungho Ryu, Miguel Cainzos-Achirica, Min-Jung Kwon, Yiyi Zhang, Yuni Choi, Jiin Ahn, Sanjay Rampal, Di Zhao, Roberto Pastor-Barriuso, Mariana Lazo, Hocheol Shin, Juhee Cho, Eliseo Guallar

**Affiliations:** 1 Departments of Epidemiology and Medicine, and Welch Center for Prevention, Epidemiology, and Clinical Research, Johns Hopkins University Bloomberg School of Public Health, Baltimore, Maryland USA; 2 Center for Cohort Studies, Total Healthcare Center, Kangbuk Samsung Hospital, Sungkyunkwan University, School of Medicine, Seoul, Republic of Korea; 3 Department of Health Sciences and Technology, Samsung Advanced Institute for Health Sciences and Technology, Sungkyunkwan University, Seoul, Republic of Korea; 4 Department of Occupational and Environmental Medicine, Kangbuk Samsung Hospital, Sungkyunkwan University, School of Medicine, Seoul, Republic of Korea; 5 Ciccarone Center for the Prevention of Heart Disease, Department of Cardiology, Johns Hopkins Medical Institutions, Baltimore, MD USA; 6 Department of Laboratory Medicine, Kangbuk Samsung Hospital, Sungkyunkwan University, School of Medicine, Seoul, South Korea; 7 Department of Social and Preventive Medicine, Julius Centre University of Malaya, Faculty of Medicine, University of Malaya, Kuala, Lumpur Malaysia; 8 National Center for Epidemiology, Carlos III Institute of Health and Consortium for Biomedical Research in Epidemiology and Public Health (CIBERESP), Madrid, Spain; 9 Department of Family Medicine, Kangbuk Samsung Hospital and Sungkyunkwan University School of Medicine, Seoul, Republic of Korea

## Abstract

The role of hepatitis virus infection in glucose homeostasis is uncertain. We examined the associations between hepatitis B virus (HBV) or hepatitis C virus (HCV) infection and the development of diabetes in a cohort (N = 439,708) of asymptomatic participants in health screening examinations. In cross-sectional analyses, the multivariable-adjusted odds ratio for prevalent diabetes comparing hepatitis B surface antigen (HBsAg) (+) to HBsAg (−) participants was 1.17 (95% CI 1.06–1.31; *P* = 0.003). The corresponding odds ratio comparing hepatitis C antibodies (HCV Ab) (+) to HCV Ab (−) participants was 1.43 (95% CI 1.01–2.02, *P* = 0.043). In prospective analyses, the multivariable-adjusted hazard ratio for incident diabetes comparing HBsAg (+) to HbsAg (−) participants was 1.23 (95% CI 1.08–1.41; *P* = 0.007). The number of incident cases of diabetes among HCV Ab (+) participants (10 cases) was too small to reliably estimate the prospective association between HCV infection and diabetes. In this large population at low risk of diabetes, HBV and HCV infections were associated with diabetes prevalence and HBV infection with the risk of incident diabetes. Our studies add evidence suggesting that diabetes is an additional metabolic complication of HBV and HCV infection.

## Introduction

Chronic viral hepatic infections are a major threat to public health worldwide. Chronic hepatitis B virus (HBV) and hepatitis C virus (HCV) are the leading causes of cirrhosis and hepatocellular carcinoma, two conditions with increasing mortality and burden of disease especially in the developing countries^1, 2^. As the liver has a key role in glucose metabolism and adequate liver function is essential to maintain glucose homeostasis^3, 4^, diabetes may be a complication of end-stage liver disease, especially in patients with chronic HCV infection^4–7^. Since diabetes is another major concern in public health, it is very important to establish if chronic viral hepatitis is associated with an increased risk of diabetes prior to the development of end-stage liver disease.

The association between positive serology for hepatitis B surface antigen (HBsAg) and incident diabetes has been studied in only a few longitudinal studies^5, 8, 9^, that found no significant associations. Cross-sectional studies have also shown no association between positive serology for HBsAg and diabetes^10, 11^, although the number of participants in these studies was small and they had varying degrees of HBV-related liver disease. On the other hand, the presence of HCV antibodies (HCV Ab) appears to increase the risk of both incident and prevalent diabetes^6, 7, 10–14^, although many of these studies were not prospective^6, 7, 11, 14^, had small sample sizes^7, 12, 13^, or included cases with liver cirrhosis which, by itself, can increase the risk of diabetes^14^. We thus examined the prospective association between HBsAg (+) or HCV Ab (+) and incident diabetes in a large cohort of asymptomatic subjects who participated in health screening examinations. In addition to the prospective associations, we also evaluated the cross-sectional associations of hepatitis virus infection and prevalent diabetes at baseline for comparability with previous studies.

## Materials and Methods

### Study population

The Kangbuk Samsung Health Study is a cohort study of all adults who underwent a comprehensive annual or biennial health screening exam at the Kangbuk Samsung Hospital Total Healthcare Screening Centers in Seoul and Suwon, South Korea^15, 16^. Regular health screening exams are mandated for employees by the South Korea Industrial Safety and Health Law. Over 80% of participants in the study were employees in a company that contracted the routine health screening exams with the Kangbuk Samsung Hospital or their relatives. The rest of participants voluntarily purchased health screening exams.

Our study had two components. First, we conducted a cross-sectional analysis of the association between HBsAg or HCV Ab and prevalent diabetes among all participants 18 to 70 years of age who underwent a comprehensive screening exam between January 1, 2002 and December 31, 2013 (*n* = 453,956). For this analysis, we excluded participants with ultrasonographic evidence of malignancy in the liver (*n* = 289), history of liver surgery or liver transplantation (*n* = 126), and history of any cancer, including hepatocellular carcinoma (*n* = 6,271). We further excluded participants with missing data on fasting glucose (*n* = 17), HBsAg (*n* = 173) or HCV Ab (*n* = 7,533). The final sample size included in the cross-sectional analysis was 439,708 (237,662 men and 202,046 women). Second, we conducted a cohort analysis of the association between HBsAg or HCV Ab and the development of incident diabetes among the 219,448 study participants (127,985 men and 91,463 women) without prevalent diabetes at baseline who had at least one follow-up screening exam prior to December 31, 2013.

We have obtained informed consent for participation from study participants starting year 2010. The Institutional Review Board (IRB) of the Kangbuk Samsung Hospital, which approved the study, waived the requirement for informed consent as we used only de-identified data that were collected as part of routine health screening exams. All methods were performed according to the local guidelines and regulations.

### Measurements

At each screening visit, study participants provided information on medical history, family history, use of medication, smoking habits, alcohol consumption, physical activity, and education level using a standardized self-administered questionnaire. Smoking status was categorized as never, former, and current. Alcohol intake was estimated in g/day and was categorized to none, moderate (≤30 g/day for males, ≤20 g/day for females), and high (>30 g/day for males, >20 g/day for females). Vigorous physical activity was categorized as <3 or ≥3 days per week. Education level was categorized as less than or equal to 12 years of education or more than 12 years of education. Participants who reported any first-degree relative with diabetes were classified as having a family history of diabetes. Anthropometric parameters and blood pressure were measured by trained staff members. Body mass index (BMI) was calculated as weight in kilograms divided by height in meters squared (kg/m^2^).

Blood specimens were sampled from the antecubital vein after at least 10 hours of fasting. Liver enzymes, including aspartate aminotransferase (AST), alanine aminotransferase (ALT), and gamma-glutamyl transferase (GGT), and serum fasting glucose were measured using Bayer Reagent Packs on an automated chemistry analyzer (Advia 1650TM Autoanalyzer; Bayer Diagnostics, Medfeld, MA, USA) between 2002 and February 2010 at the Seoul center and between 2002 and September 2006 at the Suwon center, and using a Modular Analytics D2400 (Roche Diagnostics, Tokyo, Japan) afterwards. The presence of HBsAg and HCV Ab was determined using immunoradiometric assays (Radim, Via del Mare, Italy) in the Seoul center from 2002 to 2009 and in the Suwon center from 2002 to 2006, and using electrochemiluminescent immunoassays (Modular E170; Roche Diagnostics) since 2010 in the Seoul center and since 2007 in the Suwon center. Diabetes was defined as the presence of self-reported physician diagnosis, self-reported current use of insulin or other hypoglycemic agent, or fasting serum glucose ≥126 mg/dl. The Laboratory Medicine Department at Kangbuk Samsung Hospital has been accredited by the Korean Society of Laboratory Medicine (KSLM) and the Korean Association of Quality Assurance for Clinical Laboratories (KAQACL). The laboratory also participates in the survey proficiency testing provided by the College of American Pathologists (CAP).

Abdominal ultrasonography was performed by experienced radiologists with a Logic Q700 MR 3.5-MHz transducer (GE, Milwaukee, WI, USA). A diagnosis of fatty liver disease on ultrasonography was based on the presence of diffuse hyperechoic parenchyma compared to that of the kidney or spleen^17^. A diagnosis of cirrhosis on ultrasonography was based on the presence of coarse and inhomogeneous parenchyma, caudate hypertrophy, surface nodularity, signs of portal hypertension, or regenerative nodules.

### Statistical analysis

Demographic characteristics, liver function profiles, serum fasting glucose level and the presence of hypertension, diabetes, and fatty liver disease at baseline visit were compared by HBsAg and HCV Ab status. Categorical variables were summarized as number (proportion), and continuous variables as mean (standard deviation) or median (interquartile range). Among the continuous variables compared, alcohol intake, ALT, AST, and GGT exhibited non-symmetrical distribution. Comparisons were made using a chi-square test, Student’s t-test, or Kruskal-Wallis test, as needed.

For cross-sectional analyses, we calculated odds ratios and 95% confidence intervals (CI) for prevalent diabetes by HBsAg or HCV Ab status using logistic regression. We used 3 models with progressive degrees of adjustment. Model 1: adjusted for age, sex, and center; Model 2: further adjusted for cigarette smoking, alcohol intake, education, physical activity and BMI; and Model 3: further adjusted for the presence of fatty liver disease on ultrasound.

For prospective analyses, we examined the association between HBsAg or HCV Ab status and incident diabetes. We restricted these analyses to participants without diabetes at baseline. For each individual, follow up extended from baseline until the development of diabetes or until the last screening visit. Because the exact date of the onset of diabetes occurs between two screening visits and is unknown, we used a parametric proportional hazards model to take into account this type of interval censoring (*stpm* command in Stata)^18^. The baseline hazard function was parametrized with restricted cubic splines in log time with four degrees of freedom. Hazard ratios and 95% CI for incident diabetes by HBsAg or HCV Ab status were obtained from 3 models: Model 1: adjusted for age, sex, and center; Model 2: further adjusted for cigarette smoking, alcohol intake, education, physical activity, BMI, and presence of fatty liver disease on ultrasound; and Model 3: further adjusted for the baseline fasting glucose level.

There were 35 participants who were HBsAg (+) and HCV Ab (+) at baseline, among 439,708 participants included in the cross-sectional analyses. Among 219,448 participants who were included in the prospective analyses of the study, there were 18 participants who were positive for both HBsAg and HCV antibody. Because the concomitant presence of HBsAg and HCV Ab may affect the development of diabetes, the same cross-sectional and prospective analyses were performed in a population restricted to a single exposure of HBsAg or HCV Ab.

All statistical analyses were performed with STATA version 12.0 (StataCorp LP, College Station, Texas). P values reported in the study are two-sided and P values < 0.05 were considered statistically significant.

### Data availability

The data that support the findings of this study are available from the Kangbuk Samsung Health Study and the corresponding authors upon request. The data are not publicly available as we do not have IRB approval for distribution of the data.

## Results

### Cross-sectional analysis

The cross-sectional analysis included 439,708 participants with a mean (SD) age of 39.4 (9.8) years and 54.1% of the population was male (Table 1). The prevalence of HBsAg (+) and HCV Ab (+) participants was 3.8 and 0.2%, respectively. Thirty-five participants were positive for both HBsAg and HCV Ab at baseline. HBsAg (+) and HCV Ab (+) participants were both more likely to be older, to have a higher BMI and higher levels of liver enzymes, and to consume less alcohol. The prevalence of fatty liver disease was higher in seropositive participants than in seronegative participants.

**Table 1:** Participant characteristics by hepatitis virus infection at baseline in cross-sectional analysis (*n* = 439,708).

Characteristics	Overall	Hepatitis B virus infection		*P* value	Hepatitis C virus infection		*P* value
		HBsAg (−)	HBsAg (+)		HCV Ab (−)	HCV Ab (+)	
Number (%)	439,708	423,001 (96.2)	16,707 (3.8)		438,924 (99.8)	784 (0.2)	
Age, years^*^	39.4 (9.8)	39.4 (9.9)	40.1 (9.2)	<0.001	39.4 (9.8)	48.7 (11.6)	<0.001
Men, %	54.1	53.8	61.2	<0.001	54.1	48.7	0.003
Current smoker, %	25.2	25.2	27.1	<0.001	25.2	24.4	<0.001
Alcohol intake, g/day^†^	5 (0–15)	5 (0–15)	3 (0–13)	<0.001	5 (0–15)	3 (0–14)	<0.001
Vigorous exercise, %^‡^	15.1	15.1	16.2	<0.001	15.1	20.2	<0.001
12+ years of education, %^§^	49.4	49.4	49.2	0.63	49.4	33.2	<0.001
BMI, kg/m^2*^	23.4 (3.2)	23.3 (3.2)	23.6 (3.2)	<0.001	23.4 (3.2)	23.9 (3.1)	<0.001
ALT, U/l^†^	20 (14–29)	20 (14–29)	26 (19–39)	<0.001	20 (14–29)	30 (18–50)	<0.001
AST, U/l^†^	21 (18–26)	21 (18–26)	25 (21–32)	<0.001	21 (18–26)	29 (22–41)	<0.001
GGT, U/l^†^	19 (12–34)	19 (12–34)	20 (13–35)	<0.001	19 (12–34)	23 (15–42)	<0.001
Glucose, mg/dl^*^	94.6 (16.4)	94.7 (16.3)	94.2 (17.0)	<0.001	94.6 (16.3)	99.6 (26.0)	<0.001
Prevalent diabetes, %	3.8	3.8	3.8	0.60	3.7	8.7	<0.001
Family history of DM, %	14.7	14.8	13.0	<0.001	14.7	13.7	0.39
USG Fatty liver disease, %	26.5	26.7	23.0	<0.001	26.6	21.3	0.001

Values are *means (standard deviation), ^†^medians (interquartile range), or percentages. ^‡^≥3 times per week. Abbreviations: ALT, alanine aminotransferase; AST, aspartate aminotransferase; BMI, body mass index; DM, diabetes mellitus; GGT, gamma-glutamyl transferase; USG, ultrasonography.

The prevalence of diabetes was 3.8% (*n* = 16,485). Compared to participants without diabetes, those with diabetes were more likely to be older, male, and current smokers. Also, participants with diabetes at baseline had higher proportion of family history of diabetes and fatty liver disease (Supplement Table 1) compared to those without diabetes. Participants with diabetes also had higher levels of BMI and liver enzymes and a lower frequency of education level more than 12 years.

The crude (unadjusted) prevalence of diabetes was similar in participants with and without HBsAg (3.8% in both groups). After adjusting for age, sex, center, smoking, alcohol intake, education, physical activity, BMI, and presence of fatty liver, the odds ratio for prevalent diabetes comparing HBsAg (+) to HBsAg (−) participants was 1.17 (95% CI 1.06–1.31; *P* = 0.003; Table 2). The odds ratio was unchanged after excluding participants with cirrhosis diagnosed using ultrasound (*n* = 160; odds ratio 1.17, 95% CI 1.05–1.30, *P* = 0.004).

**Table 2:** Odds ratios for prevalent diabetes by hepatitis virus infection at baseline (*n* = 439,708).

Hepatitis virus infection	Prevalent diabetes N (%)	Model 1 OR (95% CI)	Model 2 OR (95% CI)	Model 3 OR (95% CI)
**HBsAg**				
Negative	15,846 (3.8)	1.00 (reference)	1.00 (reference)	1.00 (reference)
Positive	639 (3.8)	0.98 (0.90–1.07)	1.03 (0.93–1.15)	1.17 (1.06–1.31)
***P*** **Value**		0.68	0.52	0.003
**HCV Ab**				
Negative	16,417 (3.7)	1.00 (reference)	1.00 (reference)	1.00 (reference)
Positive	68 (8.7)	1.07 (0.82–1.39)	1.14 (0.81–1.60)	1.43 (1.01–2.02)
***P*** **Value**		0.62	0.47	0.043

Model 1: adjusted for age, sex, and center; Model 2: further adjusted for smoking (never, former and current), alcohol (none, moderate and high), education (≤12 years or >12 years of education), physical activity (<3 times/week and ≥3 times/week), and BMI (continuous); Model 3: further adjusted for presence of fatty liver disease.

The unadjusted prevalence of diabetes was higher in HCV Ab (+) participants compared to HCV Ab (−) participants (8.7% vs. 3.7%, *P* < 0.001). In the multivariable-adjusted model (Model 3), the odds ratio for prevalent diabetes comparing HCV Ab (+) to HCV Ab (−) participants was 1.43 (95% CI 1.01–2.02, *P* = 0.043; Table 2). After excluding participants with cirrhosis (*n* = 160), the odds of prevalent diabetes was 1.4 times higher in HCV Ab (+) participants compared to HCV Ab (−) participants (95% CI 0.98–1.98, *P* = 0.06).

When the study population was restricted to HCV Ab (−) participants (*n* = 438,924), HBsAg (+) participants had 18% higher odds (95% CI 1.06–1.31, *P* = 0.003) of prevalent diabetes compared to HBsAg (−) counterparts (Supplement Table 2) in the fully-adjusted model. In a study population restricted to HBsAg (−) participants (*n* = 423,001), the adjusted odds ratio of prevalent diabetes between HCV Ab (+) and HCV Ab (−) participants was 1.51 (95% CI 1.06–2.13, *P* = 0.02).

### Prospective analysis

The characteristics of the 219,448 participants included in the prospective analyses were similar to those in the cross-sectional analysis (Supplement Table 3). The average duration of follow-up was 4.8 years (maximum follow-up 11.7 years). We observed 7,491 incident cases of diabetes over 1,059,666.3 person-years of follow-up. Most risk factors for incident cases of diabetes were similar to those for prevalent cases. Of note, although all participants had within-normal range fasting glucose level (<126 mg/dl), those with higher baseline levels of glucose were more likely to develop incident diabetes (Table 3).

**Table 3:** Baseline participant characteristics by incidence of diabetes in cohort analysis (*n* = 219,448).

Characteristics	Incident diabetes		*P* value
	No	Yes	
Number	211,957	7,491	
Age, years^*^	37.4 (7.6)	41.5 (8.5)	<0.001
Men, %	57.6	78.7	<0.001
Current smoker, %	26.9	39.1	<0.001
Alcohol intake, g/day^†^	5 (0–14)	8 (0–19)	<0.001
Vigorous exercise, %^‡^	14.8	17.2	<0.001
12+ years of education, %^§^	78.1	69.7	<0.001
BMI, kg/m^2*^	23.2 (3.1)	25.7 (3.2)	<0.001
ALT, U/l^†^	20 (14–29)	31 (21–48)	<0.001
AST, U/l^†^	21 (18–26)	26 (21–33)	<0.001
GGT, U/l^†^	19 (12–32)	37 (22–63)	<0.001
Glucose, mg/dl^*^	92.0 (8.3)	104.4 (10.9)	<0.001
Family history of DM, %	14.0	22.9	<0.001
USG Fatty liver disease, %	23.8	58.5	<0.001
HBsAg (+), %	4.0	4.0	0.71
HCV Ab (+), %	0.1	0.1	0.91

Values are *means (standard deviation), ^†^medians (interquartile range), or percentages. ^‡^≥3 times per week. Abbreviations: ALT, alanine aminotransferase; AST, aspartate aminotransferase; BMI, body mass index; DM, diabetes mellitus; GGT, gamma-glutamyl transferase; USG, ultrasonography.

The number of incident cases of diabetes among 8,694 HBsAg (+) participants and among 210,754 HBsAg (−) participants were 303 and 7,188, respectively (Table 4). In models adjusted for age, sex, center, smoking, alcohol intake, education, physical activity, BMI, presence of fatty liver and fasting glucose levels at baseline, the hazard ratio for incident diabetes comparing HBsAg (+) to HbsAg (−) participants was 1.20 (95% CI 1.05–1.37; *P* = 0.007). After excluding participants with cirrhosis diagnosed at baseline using ultrasound (*n* = 57), the multivariable-adjusted hazard ratio was 1.20 (95% CI 1.05–1.37, *P* = 0.007).

**Table 4:** Hazard ratios for incident diabetes by hepatitis virus infection status (*n* = 219,448).

Hepatitis virus infection	No. of incident cases	Person-years	Model 1 HR (95% CI)	Model 2 HR (95% CI)	Model 3 HR (95% CI)
**HBsAg**					
Negative	7,188	1,016,231.2	1.00 (reference)	1.00 (reference)	1.00 (reference)
Positive	303	43,435.1	0.91 (0.81–1.02)	1.07 (0.94–1.22)	1.20 (1.05–1.37)
***P*** **Value**			0.10	0.31	0.007
**HCV Ab**					
Negative	7,481	1,058,402.5	1.00 (reference)	1.00 (reference)	1.00 (reference)
Positive	10	1,263.8	0.77 (0.42–1.44)	0.72 (0.30–1.73)	0.75 (0.31–1.81)
***P*** **Value**			0.42	0.46	0.52

Model 1: adjusted for age, sex, and center; Model 2: further adjusted for smoking (never, former and current), alcohol (none, moderate and high), education (≤12 years or >12 years of education), physical activity (<3 times/week and ≥3 times/week), BMI (continuous), and presence of fatty liver disease; Model 3: further adjusted for initial fasting glucose level.

The number of incident cases of diabetes among 283 HCV Ab (+) participants and among 219,165 HCV Ab (−) participants were 10 and 7,481 (Table 4). The multivariable-adjusted hazard ratio for incident diabetes comparing HCV Ab (+) and HCV Ab (−) participants was 0.75 (95% CI 0.31–1.81; *P* = 0.52). The hazard ratio was unchanged after excluding participants with cirrhosis diagnosed using ultrasound at baseline.

In a study population restricted to HCV Ab (−) participants (*n* = 219,165), HBsAg (+) participants had 20% higher hazard (95% CI 1.05–1.37, *P* = 0.008) of incident diabetes compared to HBsAg (−) participants (Supplement Table 4) in the multivariable-adjusted model. When restricted to HBsAg (−) participants (*n* = 210,754), the adjusted hazard ratio for incident diabetes between HCV Ab (+) and HCV Ab (−) participants was 0.77 (95% CI 0.32–1.86, *P* = 0.57).

Since serological evaluation of HBV and HCV infection was included as a routine part of the blood work in all health exams in our cohort, we could also determine participants who maintained their serologic status over follow-up. Among 8,694 participants who were HBsAg (+) at baseline, there were 535 participants who converted to HBsAg (−) over time, and among 210,754 participants who were who were initially HBsAg (−), 28 participants converted to HBsAg (+). Among 283 participants who were HCV Ab (+) at baseline, 59 of them converted to HCV Ab (−), and among 219,165 participants who were initially HCV Ab (−), 77 converted to HCV Ab (+). In fully-adjusted models restricted to participants who did not change serologic status over time, the hazard ratio for incident diabetes comparing HBsAg (+) to HBsAg (−) participants was 1.24 (95% CI 1.08–1.43; P = 0.002), and the corresponding hazard ratio for comparing HCV Ab (+) to HCV Ab (−) participants was 0.78 (95% CI 0.32–1.86; P = 0.57).

## Discussion

In this large study of healthy men and women, we found a cross-sectional association of HBV and HCV infection with the prevalence of diabetes. The association was stronger for HCV compared to HBV. The associations were evident even after excluding participants with cirrhosis on ultrasound exam. For HBV, the association was also observed in a prospective analysis of participants who did not have diabetes at baseline. We did not find an association between HCV infection and the risk of diabetes in prospective studies, but the number of incident cases among HCV Ab (+) was too small to obtain reliable risk estimates. Our study adds novel evidence on a prospective association of HBV infection and the risk of incident diabetes and adds to the evidence that hepatitis virus infection is linked to the development of glucose metabolism abnormalities.

The association between HBV infection and diabetes has been controversial. The prevalence of diabetes was higher in HBsAg (+) compared to HBsAg (−) patients in some studies^19–21^, but not in others^7, 10^. In a 10-year prospective study restricted to subjects without cirrhosis, HBV infection was not associated with the incidence of diabetes or glucose intolerance^8^, but this study was limited by a small sample size. In the present study, the large sample size, the use of careful laboratory methods to assess incident diabetes, and the ability to adjust for multiple baseline covariates, including the presence of fatty liver disease, allowed us to identify a clear association between HBsAg and diabetes in both cross-sectional and incident analyses.

With respect to HCV, our finding of an association between HCV Ab (+) and diabetes are consistent with most previous studies. In a meta-analysis of 17 studies comparing HCV-infected participants to HCV non-infected participants, the pooled OR for diabetes was 1.68^22^. In our study, the cross-sectional association between HCV infection and diabetes was identified even though the prevalence of HCV Ab (+) participants in our study was lower compared to that of other studies and the overall risk of diabetes in our study population was low because of young age and low BMI. This finding thus confirms that HCV virus infection can be a risk factor for diabetes even in low risk populations.

Given the pivotal role of the liver in glucose metabolism^3, 4^, the presence of severe hepatic diseases, such as liver cirrhosis and hepatocellular carcinoma, leads to dysregulation in glucose homeostasis^4, 17, 23^. The mechanisms underlying the disruption in glucose metabolism in the context of viral hepatitis without cirrhosis, however, is unclear^14, 24–26^. In one study, patients with either chronic HBV or HCV infection with elevated liver enzymes showed a strong association between the extent of fibrosis in liver biopsies and diabetes, but without differences between HBV and HCV infection^21^. An Italian group also found cirrhosis and age to be the only two factors independently associated with the presence of diabetes in patients with chronic HCV infection^27^.

Other mechanisms have been suggested to explain altered glucose metabolism in HCV infection. In experimental studies HCV can directly disturb the insulin signaling cascade in infected hepatocytes^28, 29^, which may be driven by genotype-specific pathways^30^. HCV may also interfere with glucose metabolism indirectly by causing peripheral insulin resistance in muscle tissue of chronic HCV patients^31^. The effect of HBV infection on the development of diabetes is less understood and has been mostly attributable to HBV-related cirrhosis^32^. Additional research is needed to understand the mechanisms involved in altered glucose metabolism in HBV and HCV patients.

Although we had a large sample size and implemented both cross-sectional and longitudinal study designs, there are a few limitations. First, the number of participants with HCV Ab in the study was relatively small (0.18%). The prevalence of HCV Ab (+) in the general Korean adult population ranges from 0.5 to 1.0% with the highest prevalence in those over 60 years of age^33, 34^. The prevalence in of HCV Ab (+) in Korea is low by global standards (global estimates of prevalence 2.35%)^35^. In our study, we observed a clear association between HCV Ab (+) and prevalent diabetes even though our population was at low risk of both HCV and diabetes and was comprised of relatively young, healthy, and low BMI participants. Unfortunately, we were unable to confirm this association in prospective analysis because there were only 10 cases of incident diabetes among HCV Ab (+) participants during follow-up (The power of our study to detect relative risks for diabetes of 1.10, 1.20, and 1.30 when comparing HCV Ab (+) to HCV Ab (−) participants was 6.1, 9.4, and 14.8%, respectively).

Second, diabetes was defined based on self-reported medical history, medication history, and a single measurement of fasting serum glucose, which is a commonly used definition in epidemiologic studies but differs from clinical diagnostic criteria for diabetes^36^. Our definition will introduce some degree of misclassification of the outcome status, but this type of misclassification is likely to be non-differential and, therefore, would induce an attenuation of the association. In addition, we cannot distinguish between cases of type 1 and type 2 diabetes in our data. However, we expect that most new cases of diabetes occurring in this cohort are type 2 diabetes because the participants in this cohort were adults (mean age 39.4 years) and we excluded participants with diabetes at baseline.

Third, study participants were followed for a maximum of 12 years (average duration of follow-up 4.8 years). It is possible that the full effects of hepatitis virus infection on diabetes and glucose metabolism take longer to manifest in chronically infected patients. Further longitudinal studies with longer duration of follow-up are needed to fully characterize the effects of hepatitis virus infection on the risk of diabetes. Fourth, although ultrasound is a well-established, widely-used non-invasive modality to diagnose liver cirrhosis in population studies, its sensitivity and specificity to detect liver cirrhosis are imperfect (sensitivity 54–84%, specificity 80–100%, positive predictive value 68–87%, and negative predictive value 36–42%)^37, 38^. Therefore, some cases of liver cirrhosis may have been misdiagnosed and included in our sensitivity analyses. Fifth, the level and frequency of physical activity was measured using a self-reported questionnaire, which may not be the most accurate method to assess physical activity and can be subject to some misclassification. Finally, the study was conducted among participants attending routine health exams and may not be generalizable to other populations or ethnic groups.

In conclusion, in this large study of men and women at low risk of diabetes, we found that serologic evidence of HBV and HCV infection was associated with the prevalence of diabetes. In addition, HBV infection was associated with the risk of incident diabetes in prospective analyses, but we could not reliably evaluate the prospective association between HCV infection and diabetes due to the small number of infected participants. Our studies add to the growing body of evidence suggesting that diabetes is an additional metabolic complication of HBV and HCV infection.

## Electronic supplementary material

Supplementary Information
