# Supplementary material for: Hepatitis B and C virus infection and diabetes mellitus: A cohort study
Source: Sci Rep. 2017 Jul 4;7:4606. doi: 10.1038/s41598-017-04206-6 (PMC5496892; doi:10.1038/s41598-017-04206-6)
Supplement: Supplementary file 1 — Supplementary Information [file 41598_2017_4206_MOESM1_ESM.pdf]

Hepatitis B and C virus infection and diabetes mellitus: A cohort study

Yun Soo Hong, MD,[1*] Yoosoo Chang, MD, PhD, [2,3,4*] Seungho Ryu, MD, PhD, [2,3,4] Miguel Cainzos-Achirica, MD, [1,5] Min-Jung Kwon, MD, PhD, [2,6] Yiyi Zhang, PhD,[1] Yuni Choi, BS,[2] Jiin Ahn, MSPH,[2] Sanjay Rampal, MD,[1,7] Di Zhao, MHS,[1] Roberto Pastor-Barriuso, PhD,[8] Mariana Lazo, MD, PhD,[1] Hocheol Shin, MD, PhD,[9] Juhee Cho, PhD,[1,2,3] Eliseo Guallar, MD, DrPH.[1]

[1] Departments of Epidemiology and Medicine, and Welch Center for Prevention, Epidemiology, and Clinical Research, Johns Hopkins University Bloomberg School of Public Health. Baltimore, Maryland, USA.

[2] Center for Cohort Studies, Total Healthcare Center, Kangbuk Samsung Hospital, Sungkyunkwan University, School of Medicine, Seoul, Republic of Korea

[3] Department of Health Sciences and Technology, Samsung Advanced Institute for Health Sciences and Technology, Sungkyunkwan University, Seoul, Republic of Korea

[4] Department of Occupational and Environmental Medicine, Kangbuk Samsung Hospital, Sungkyunkwan University, School of Medicine, Seoul, Republic of Korea

[5] Ciccarone Center for the Prevention of Heart Disease, Department of Cardiology, Johns Hopkins Medical Institutions, Baltimore (MD), USA.

[6] Department of Laboratory Medicine, Kangbuk Samsung Hospital, Sungkyunkwan University, School of Medicine, Seoul, South Korea.

[7] Department of Social and Preventive Medicine, Julius Centre University of Malaya, Faculty of Medicine, University of Malaya. Kuala Lumpur, Malaysia.

[8] National Center for Epidemiology, Carlos III Institute of Health and Consortium for Biomedical Research in Epidemiology and Public Health (CIBERESP), Madrid, Spain

[9] Department of Family Medicine, Kangbuk Samsung Hospital and Sungkyunkwan University School of Medicine. Seoul, Republic of Korea

***** Drs. Hong and Chang contributed equally as co-first authors of this paper.

Supplement Table 1. Participant characteristics by prevalence of diabetes at baseline in cross-sectional analysis (*n* = 439,708).

Characteristics	Prevalent diabetes		*P* value
	No	Yes	
Number	423,223	16,485	
Age, years*	39.0 (9.5)	50.6 (10.5)	< 0.001
Men, %	53.5	69.0	< 0.001
Current smoker, %	25.1	29.8	< 0.001
Alcohol intake, g/day†	5 (0-15)	6 (0-23)	< 0.001
Vigorous exercise, %‡	14.9	21.9	< 0.001
12+ years of education, %§	50.0	32.7	< 0.001
BMI, kg/m^2*	23.3 (3.2)	25.4 (3.3)	< 0.001
ALT, U/l†	20 (14-29)	28 (20-42)	< 0.001
AST, U/l†	21 (18-26)	25 (20-33)	< 0.001
GGT, U/l†	19 (12-33)	35 (21-64)	< 0.001
Glucose, mg/dl*	92.6 (8.9)	148.1 (46.3)	< 0.001
Family history of DM, %	14.1	31.8	< 0.001
USG Fatty liver disease, %	25.2	63.1	< 0.001
HBsAg (+), %	3.8	3.9	0.60
HCV Ab (+), %	0.2	0.4	< 0.001

Values are *means (standard deviation), †medians (interquartile range), or percentages.
‡ ≥ 3 times per week.
Abbreviations: ALT, alanine aminotransferase; AST, aspartate aminotransferase; BMI, body mass index; DM, diabetes mellitus; GGT, gamma-glutamyl transferase; USG, ultrasonography.

Supplement Table 2. Odds ratios for prevalent diabetes among HCV Ab-negative participants and among HBsAg-negative participants.

**Hepatitis virus infection**	**Prevalent diabetes N (%)**	**Model 1 OR (95% CI)**	**Model 2 OR (95% CI)**	**Model 3 OR (95% CI)**
**HBsAg*** (*n* = 438,924)				
Negative	15,778 (3.7)	1.00 (reference)	1.00 (reference)	1.00 (reference)
Positive	639 (3.8)	0.99 (0.91-1.07)	1.04 (0.93-1.15)	1.18 (1.06-1.31)
***P* Value**		0.77	0.50	0.003
**HCV Ab**† (*n* = 423,001)				
Negative	15,846 (3.8)	1.00 (reference)	1.00 (reference)	1.00 (reference)
Positive	68 (9.1)	1.11 (0.85-1.44)	1.19 (0.84-1.68)	1.51 (1.06-2.13)
***P* Value**		0.44	0.33	0.02

*Analyses restricted to participants who are HCV Ab-negative; †Analyses restricted to participants who are HBsAg-negative.

Model 1: adjusted for age, sex, and center; Model 2: further adjusted for smoking (never, former and current), alcohol (none, moderate and high), education (≤ 12 years or > 12 years of education), physical activity (< 3 times/week and ≥ 3 times/week), and BMI (continuous); Model 3: further adjusted for presence of fatty liver disease.

Supplement Table 3. Baseline participant characteristics by hepatitis virus infection in cohort analysis (*n* = 219,448).

**Characteristics**	**Overall**	**Hepatitis B virus infection**		***P* value**	**Hepatitis C virus infection**		***P* value**
		**HBsAg (-)**	**HBsAg (+)**		**HCV Ab (-)**	**HCV Ab (+)**	
Number (%)	219,448	210,754 (96.0)	8,694 (4.0)		219,165 (99.9)	283 (0.1)	
Age, years*	37.5 (7.6)	37.4 (7.6)	38.2 (7.3)	< 0.001	37.5 (7.6)	43.0 (10.2)	< 0.001
Men, %	58.3	58.2	65.6	< 0.001	58.3	50.2	0.005
Current smoker, %	27.3	27.3	28.8	< 0.001	27.3	25.8	0.92
Alcohol intake, g/day†	5 (0-15)	5 (0-15)	3 (0-12)	< 0.001	5 (0-15)	3 (0-12)	< 0.001
Vigorous exercise, %‡	14.9	14.8	16.3	< 0.001	14.9	20.1	0.005
12+ years of education , %§	51.5	51.5	52.4	0.004	51.5	40.6	< 0.001
BMI, kg/m^2*	23.3 (3.1)	23.3 (3.1)	23.6 (3.1)	< 0.001	23.3 (3.1)	23.6 (3.2)	0.08
ALT, U/l†	20 (15-30)	20 (14-29)	27 (19-39)	< 0.001	20 (15-30)	25 (17-43)	< 0.001
AST, U/l†	22 (18-26)	21 (18-26)	25 (21-32)	< 0.001	22 (18-26)	26 (20-35)	< 0.001
GGT, U/l†	19 (12-33)	19 (12-33)	20 (13-34)	< 0.001	19 (12-33)	20 (13-32)	0.52
Glucose, mg/dl*	92.4 (8.7)	92.4 (8.7)	91.8 (8.7)	< 0.001	92.4 (8.7)	92.9 (9.2)	0.35
Family history of DM, %	14.3	14.4	12.7	< 0.001	14.3	14.5	0.93
USG Fatty liver disease, %	24.9	25.1	22.1	< 0.001	25.0	19.4	0.03

Values are *means (standard deviation), †medians (interquartile range), or percentages.

‡ ≥ 3 times per week.

Abbreviations: ALT, alanine aminotransferase; AST, aspartate aminotransferase; BMI, body mass index; DM, diabetes mellitus; GGT, gamma-glutamyl transferase; USG, ultrasonography.

Supplement Table 4. Hazard ratios for incident diabetes among HCV Ab-negative participants and among HBsAg-negative participants.

Hepatitis virus infection	No. of incident cases	Person-years	Model 1 HR (95% CI)	Model 2 HR (95% CI)	Model 3 HR (95% CI)
**HBsAg*** (*n* =219,165)					
Negative	7179	1,015,039.3	1.00 (reference)	1.00 (reference)	1.00 (reference)
Positive	302	43,363.2	0.90 (0.81-1.01)	1.07 (0.93-1.22)	1.20 (1.05-1.37)
***P* Value**			0.09	0.34	0.008
**HCV Ab**† (*n* = 210,754)					
Negative	7179	1,015,039.3	1.00 (reference)	1.00 (reference)	1.00 (reference)
Positive	9	1,191.9	0.73 (0.38-1.40)	0.74 (0.31-1.78)	0.77 (0.32-1.86)
***P* Value**			0.34	0.50	0.57

*Analyses restricted to participants who are HCV Ab-negative; †Analyses restricted to participants who are HBsAg-negative.
Model 1: adjusted for age, sex, and center; Model 2: further adjusted for smoking (never, former and current), alcohol (none, moderate and high), education (≤ 12 years or > 12 years of education), physical activity (< 3 times/week and ≥ 3 times/week), BMI (continuous), and presence of fatty liver disease; Model 3: further adjusted for initial fasting glucose level.
